# Connexin 43 Loss in Endothelial Progenitors Facilitates Functional Airway Adaptation After Lung Injury

**DOI:** 10.21203/rs.3.rs-9727235/v1

**Published:** 2026-06-09

**Authors:** Maggie Dawon, Hannah F Thorndyke, Julia M Hollaway, Preeyam Patel, Bradley W Richmond, Carlyne D Cool, Edward P Manning, Jonathan A Kropski, Moumita Ghosh, Aleksandra Tata, Patrick Geraghty, Susan M Majka

**Affiliations:** National Jewish Health; National Jewish Health; National Jewish Health; Independent Researcher; Vanderbilt University Medical Center; National Jewish Health; Yale University; Vanderbilt University Medical Center; University of Colorado Anschutz Medical Campus; Duke Medical Center; SUNY Downstate Medical Center; National Jewish Health

**Keywords:** Microvascular endothelial progenitor cell (mvEPC), ATP Binding Cassette Subfamily G Member 2 (Abcg2), Connexin 43 (Gja1), alveolar-capillary network, reparative angiogenesis, fibrosis, microvascular niche, microvascular endothelial cells, emphysematous loss of structure

## Abstract

Connexin 43 mediated gap junction signaling between ABCG2^hi^ endothelial progenitor cells and alveolar epithelium governs epithelial differentiation and injury response. Here, we identify a previously unrecognized endothelial - epithelial communication axis that regulates AT2-to-AT1 transition during fibrotic repair. Disruption of ABCG2^hi^ derived endothelial Cx43 impairs canonical regeneration but activates an alternative basal cell driven repair program. These findings reveal intercellular communication as a key determinant of lung repair trajectory.

Intercellular communication mediated by gap junctions between alveolar epithelium and the capillary endothelium plays a fundamental role in the development and maintenance of the alveolar-capillary niche^1,2^, as well as responses to stress, injury and aging^1,3–5^. Connexin (Cx) expression varies along the respiratory and vascular trees. Endothelial connexins in the lung include Cx40, and Cx43, with Cx37 present in the macrovasculature^6^, while epithelial cells express Cx43, 46, 26, 32 and 37^1^. Lung fibroblasts primarily express Cx43 and 45^3^. Connexins exhibit selective affinity for one another, regulating intercellular communication between specific cell types.

Cx43 is necessary for normal morphogenesis of the alveolar-capillary unit^1^. Mice lacking Cx43 die after birth due to lung hypoplasia characterized by impaired alveolarization^2^, including reduced numbers of alveolar epithelial type I cells (AT1), increased type II cells (AT2)^2^. Cx43 is also strongly expressed in both the alveolar endothelium and epithelium (AT1/AT2) and facilitates communication between AT1 and AT2^1,3,4,7^. In humans and animal models, altered Cx43 expression has been associated with aging ^5^ and acute or chronic lung diseases, including fibrosis, chronic obstructive pulmonary disease (COPD)/emphysema, acute lung injury (ALI)/acute respiratory distress syndrome (ARDS), and asthma^8–11^. Collectively, these findings indicate that Cx43 plays a central role in alveolar epithelial differentiation and homeostasis in the alveolar-capillary unit.

Previous studies have demonstrated a link between capillary endothelial regulation of AT1/AT2 cell differentiation and function^12,13^. Gap junctional communications have been shown to occur between embryonic endothelial progenitor cells (EPC) or adult ABCG2^hi^ EPC with capillary endothelium as well as AT1 epithelium^14,15^. However, a direct intercellular communication link between the angiogenic ABCG2^hi^ EPC and response of the alveolar epithelium to injury or during disease has not been established.

ABCG2^hi^ endothelium in the alveolar capillary network are enriched for endothelial progenitor cells with colony forming unit and rigorous angiogenic potential^16–19^. To localize Cx43 expression in adult mouse lung we utilized the inducible ABCG2 lineage tracing system, mice were induced with a single low dose tamoxifen to label the cells with highest activity of the ABCG2 promoter^16^. Lung tissue was harvested post induction, and immunostaining was performed on vibratome (Figure 1F and G) and tissue sections (**Supplemental Figure 1**). Maximum intensity projections localized Cx43 to eGFP expressing ABCG2^hi^ EPC as well as distributed through the alveolar capillary unit (Figure 1F and G; **Supplemental Figure 1**).

We next used this lineage driver system to deplete Cx43 (cCx43^+/−^) in ABCG2^hi^ EPC using a floxed *Gja1* allele^20^. Mice that were heterozygous for the floxed allele were induced with a single low dose tamoxifen. Lungs were collected two days post induction and processed to form a single cell suspension and analyzed by flow cytometry to detect eGFP labeled ABCG2^hi^ EPC (Figure 1C). A significant decrease in EPC was noted in the cCx43^+/−^ mice relative to wild-type (WT) suggesting that EPC require Cx43-mediated communication with AECs to effectively sense injury and coordinate expansion and differentiation.

To assess the functional significance of Cx43 depletion in ABCG2^hi^ EPC on the alveolar – capillary unit we performed a time course of bleomycin injury induced fibrosis and repair over the course of day14 (peak fibrosis) to 3 months (repair; Figure 1D) as well as a 16 week analysis of baseline phenotype (**Supplemental Figure 4**) comparing wildtype (WT) and cCx43^+/−^ mice. The cCx43^+/−^ mice had a higher rate of survival following injury (Figure 1E). Both mouse strains demonstrated significant increases consistent with fibrosis at day 14, including increased tissue volume and vascular leak, decreased aerated lung volume (Figure 1F), inspiratory capacity (IC), compliance (Crs and Cst), and increased elastance (Ers; Figure 1G). Interestingly, the tissue volume, a three-dimensional quantitation of structural changes in tissue density, was significantly increased in the WT mice relative to cCx43^+/−^ miceat peak fibrosis (Figure 1F), illustrating a difference in the progression, composition or cell density of fibrosis conferring a survival advantage.

A corresponding significantly decrease in microvessel (<5micron) density was quantitated in the PBS vehicle control and bleomycin treated lungs at day14 relative to the PBS vehicle (Figure 1H–J), however, there was no difference between strains. Representative maximum intensity projections from the lectin perfused lungs highlighted the decreased presence of ABCG2^hi^ eGFP EPC in cCx43^+/−^ mice in PBS vehicle and following bleomycin, in contrast to the WT, where there was a visible expansion of the EPC (Figure 1K and L), suggesting the cCx43^+/−^EPC responded to injury differently than WT.

Histological analyses confirmed alterations in both composition and cell density of fibrosis indicated by the tissue volume (Figure 1F). Significant differences in alveolar tissue structure between the WT and cCx43^+/−^ mice at peak fibrosis were evident with trichrome stain (Figure 1M,N). cCx43^+/−^ mice exhibited striking peribronchiolar metaplasia, or the replacement of alveolar epithelium by an ingrowth of bronchiolar airway epithelium commonly associated with interstitial lung diseases^21^, in contrast to the WT mice that presented with typical cell hyperplasia and collagen deposition (Figure 1M,N; **Supplemental Figure 2**). This response by the cCx43^+/−^ mice suggests an adaptive repair response due to decreased Cx43 expression by ABCG2^hi^ EPC.

Typically, whether adaptive epithelial programs occur is dependent on the extent of injury, and type of epithelium damaged (AT1, AT2, Club cell etc.)^22^. Severe damage to AT2 cells, the alveolar stem cells, may impair their self-renewal and AT1 differentiation potential, triggering an adaptive airway progenitor response to initiate repair. Typical airway progenitors that contribute to remodeling and repair following profound injury have been characterized as expressing combinations of p63, SOX2 and cytokeratin 5 (Krt5), however, airway resident lineage negative epithelial progenitors have also been described^22–28^. To further characterize the metaplastic structures, immunostaining was performed to detect SOX2, surfactant protein C (SFTPC) and Cx43 (Figure 2). The ectopic basal cells in cCx43^+/−^ mice highly expressed SOX2 and Cx43 and were negative for SFTPC (Figure 2A,C,E), whereas the WT mice exhibited only a small number of SOX2 and Cx43 positive cells in the areas of fibrosis (Figure 2B,D,F).

Interestingly, based on microCT and airway physiology data from peak fibrosis on day 14 to 3 months, both the WT and cCx43^+/−^ mouse lungs underwent repair (**Supplemental Figure 3**). However, upon histological analysis it appeared that the cCx43^+/−^ mice had areas of non-resolved injury. This disparate finding has been reported in mouse models of emphysema as well as patients, where the diffusing capacity for carbon monoxide (DLCO) across the alveoli was more correlative of alveolar remodeling^29–31^. To validate these findings we employed a second transgenic model to disrupt ABCG2^hi^ EPC – alveolar epithelial communication by using Diphtheria toxin A depletion of the EPC ^17,32,33^. Histological evaluation defined the appearance of ectopic basal epithelial cells in the injured alveoli at day 14 post bleomycin (Supplemental Figure 3F&G). In the absence of injury no significant differences in vascular leak, microCT or airway physiology were detected (**Supplemental Figure 4A-E,G,I**). An increase in right ventricular systolic pressure was quantified in the absence of a change in the Fulton index, alluding to EC dysfunction and pulmonary hypertension(**Supplemental Figure 4F**). Taken together, the data suggests that alveolar epithelium, EPC, and basal epithelium communicate via Cx43 during injury, repair, that the appearance of ectopic basal cells represents a pro-repair adaptive epithelial response. The level of alveolar disrepair, undetectable by conventional analysis, may precede the development of a chronic pathology.

Such an adaptive epithelial response is characteristic of IPF when significant changes in epithelial transcriptomics^34,35^ occur in parallel to the accumulation of atypical epithelial cells in the alveolar space forming bronchiolized or honeycomb structures^35^. The ectopic basal cells are likely part of a pro-repair program following AT2 dysfunction and may transition to pathogenic if repair is stalled. To address this probability, human lung tissue was analyzed to characterize the expression of *GJA1*, the gene for Cx43, in pulmonary endothelial(EC) subpopulations using publicly available human scRNA-seq data sets with representation of control, idiopathic pulmonary fibrosis (IPF) and COPD patient samples (Figure 3A). *GJA1* expression increased in the capillary CAP1 population in IPF and less so COPD. We then analyzed the expression of *GJA1* in nonhematopoietic (CD45 negative) lung cells from an IPF dataset (Figure 3B). *GJA1* expression was increased in fibroblasts, lymphatic EC, CAP1(gCap), AT2 and AT1 cells in IPF relative to control. The expression of *GJA1* and Cx43 was quantified in isolated human ABCG2^hi^ EPC^16^. RT-PCR analysis of primary EPC demonstrated an increase in *GJA1 i*n IPF samples relative to control (Figure 3C). Cx43 expression paralleled this finding with increased levels in the IPF samples (Figure 3D).

In summary, our results demonstrate a direct impact of the alveolar endothelium on the alveolar epithelial repair program. We show that with a decrease of ABCG2^hi^ endothelial progenitor Cx43 expression or communication, the normal AT1/AT2 repair program is disrupted, triggering an adaptive epithelial response. Under these conditions, ectopic basal cells may transiently help maintain tissue structure and function while the alveolar epithelium and endothelium undergo incomplete repair, predisposing the gas exchange unit to further remodeling. Although these ectopic basal cells can provide an initial functional survival benefit, they may become pathogenic if they persist during repeated injury and concurrent stem cell depletion within the alveolar-capillary unit. Taken together, these studies demonstrate the functional significance of Cx43 mediated gap junction- communication between alveolar endothelium and epithelium, and their progenitors in the pulmonary tissue repair and pathology.

## METHODS

### Study Approval:

The Institutional Animal Care and Use Committee (IACUC) at National Jewish Health approved all animal procedures and protocols. Banked patient cell lines were obtained using IRB #9401 approved by the Vanderbilt University Medical Center IRB Committee, Nashville, TN, USA. Patients were consented under this IRB for the generation and storage of human cell lines as previously described ^16,19,36^. Abcg2-Cre^ERT2^ mice, shared by Dr. B. Sorrentino (St. Jude Children’s Research Hospital, Memphis, TN)^37^, were crossed with an enhanced green fluorescent protein (eGFP) reporter (B6.129(Cg)-Gt(ROSA)26Sor^tm4(ACTB-tdTomato,-EGFP)Luo^/J, JAX Stock No. 007676; designated mT/mG) strain to facilitate lineage tracing of adult microvascular endothelial progenitor cells (mvEPC), designated WT. WT mice were bred to floxed Connexin 43 mice (JAX Stock No. 008039; B6.129S7-*Gja1*^*tm1Dlg*^/J). All Connexin 43 mice were heterozygous (termed cCx43^+/−^) with the reporter, as they are both inserted into the Rosa 26 locus. To knockout mvEPC *in vivo*, we crossed the ABCG2 driver/reporter strain to a mouse containing a floxp stop allele regulating the expression of Diphtheria toxin A (DTA) ^17,32,33^ (JAX stock# 009669). Cre-Lox recombination for lineage tracing and transgene activation was induced through a single low dose intraperitoneal injection of 0.5 mg tamoxifen (Sigma, T5648–5G) as previously described ^16,19^. Fourteen days post tamoxifen induction, bleomycin sulfate (0.05 U/18g) or phosphate buffered saline vehicle was delivered via oropharyngeal aspiration. Lungs were collected and analyzed at day-14, the peak fibrotic response and 1.5 or 3 months post injury.

### Alterations in the lung as a result of Connexin 43 depletion in ABCG2^hi^ EPC.

A detailed description of measures of lung leak, RVSP, flexivent measures of airway physiology and microCT was performed as previously described^19,38,39^. Lung leak was measured by collecting bronchoalveolar lavage fluid (BALF) and samples were run in triplicate using an Albumin ELISA kit (Fortis Life Sciences, Waltham, MA) following the manufacturer’s protocol. To analyze changes in lung structure, lungs were inflated using 0.75% low-melt agarose (Isc Bio Express, Kaysville, UT) in phosphate buffered saline (PBS) (Fisher, Hampton, NH) and fixed in 10% Buffered Formalin (Fisher, Hampton, NH) overnight. 5μm thick paraffin sections were stained with standard hematoxylin-eosin (H&E) or Masson’s trichrome^39^. Mouse lung sections were imaged using a BZ-X810 Keyence Microscope with a 4X PlanFluor objective in Brightfield. Tiled images of the entire lung section were stitched together using the BZ-X800 Analyzer Software (1.1.2.4). Images of H&E or Masson’s trichrome stained lung sections were taken on a Nikon Eclipse 80i microscope using NIS Elements BR software (v 5.30.03).

To quantitate microvascular density (<5 micron diameter), PBS vehicle or bleomycin treated WT and cCx43^+/−^ mouse lungs were harvested at day-14 (n=6–8 per group). At harvest, mice were retro-orbitally injected with *Lycopersicon esculentum* tomato Lectin (LEL)-Dylight649 (Vector Laboratories Inc., DL-1178–1) and lungs were inflated with 2% Low Melt Agarose. The left lung lobes were vibratome sectioned to obtain 150μm PCLS. These PCLS were mounted using SlowFade Glass Soft-set Antifade Mountant with DAPI (Invitrogen, S36920). Z-stacks of the PCLS were then imaged with a Zeiss LSM900 confocal microscope. CZI files of the 3D-Airyscan Processed z-stacks were analyzed by Wimasis Image Analysis (Onimagin Technologies). Vessel density was quantitated as previously described^40^.

### Characterization of Connexin 43/*GJA1* Expression by Human Lung ABCG2^hi^ EPC.

Humanlung ABCG2^hi^ EPC were isolated from tissue explants of adult control (n=3–4 Male 57 and 21 years old and Female 66 and 32 years old), and fibrosis (n=3–4 Male 62, 64, 60 years old and Female 67 years old) and characterized as previously described ^15,16,18,19,41^. Briefly, lung tissue was digested with Type II Collagenase (Worthington, LS004202) to a single cell suspension. The resulting single cell suspension of whole lung was analyzed by flow cytometry to detect CD45^neg^ Abcg2^pos^ cells via cell surface antigen. OneComp eBeads compensation beads (Invitrogen, 01–1111-42) were used to set voltages and gating parameters. 4',6-diamidino-2-phenylindole (DAPI) was used to exclude dead cells (Invitrogen, R37606). To quantitate Connexin 43 expression in primary human mvEPC, semi-quantitative RT-PCR was performed to detect the *GJA1* gene. Primary human mvEPC were collected and RNA was extracted using the Qiagen RNeasy Mini Kit (Qiagen, 74104). The RNA was processed using the TaqMan RNA-to-Ct 1-Step Kit (Thermo Fisher, 4392938). Reactions were set up in a 96-well plate using the respective TaqMan kit and specific TaqMan gene expression assays for the target gene of interest. Fluorescence data was collected and the threshold cycle (Ct) values for each sample were determined. Gene expression levels were calculated using the delta Ct method, normalizing human *GJA1* to *GAPDH*.

Single-cell RNA-seq data reanalysis from human lung samples (GSE136831^34^, Control, IPF, COPD) were analyzed using Python (Scanpy/AnnData). Endothelial cells were subsetted and refined into standardized subtypes based on annotated cell identities. Gene expression values were normalized and log-transformed prior to analysis. Dot plots were generated to visualize ***GJA1* (Cx43)** expression across endothelial subtypes, where dot size represents the fraction of cells expressing the gene and color intensity reflects mean expression within each group. Group-wise comparisons were performed across disease conditions (Control, IPF, COPD) using consistent scaling to enable direct visualization of relative expression differences. IPF scRNA-seq reanalysis utilized IPF and control lung samples from GSE227136^42^ were queried for expression of *GJA1*. The integrated processed object was subset to IPF and control samples and exclude immune cells. *GJA1* relative expression was plotted using Scanpy (v1.9.6)^43^.

To quantitate Cx43 protein,murine lungs or primary human lungs, ABCG2^hi^ EPC were lysed with RIPA Buffer (Cell Signaling, 9806S) containing protease and phosphatase inhibitors (Thermo Fisher Scientific, 78444) and 0.5M EDTA Solution (Thermo Fisher Scientific, 1861283) to collect protein extracts. Protein concentration was determined using a Pierce BCA Protein Assay Kit (Thermo Fisher Scientific, 23227) following the manufactures’ instructions. After standardization, protein extracts were mixed with Laemmli Sample Buffer (BioRad, 1610747), resolved on 1.5 mm thick NuPAGE 4–12% Bis-Tris gels (Thermo Fisher Scientific, NP0335BOX), and transferred to PVD membranes. Blots were blocked with 5% dry milk and treated overnight at 4°C with Connexin 43 antibody (Cell Signaling, 3512S) to detect target protein. The blots were washed and treated with appropriate secondary antibody. Blots were visualized with Clarity Western ECL Substrate (BioRad, 170–5060) then imaged and analyzed on a Licor Odyssey Imager. Immunofluorescent staining and imaging was performed to lineage label eGFP-labeled lung mvEPC as described^16,17,19,40^. Additionally, 300–500μm PCLS were vibratome sectioned and were blocked using a 0.1% PBS + Triton with 10% FBS solution for 3 hrs. at room temperature. Sections were then incubated overnight at room temperature with an anti-Connexin 43 and anti-GFP (Antibodies Inc., GFP-1010) primary antibody at a concentration of 1:50 and 1:500 respectively in 0.1% PBS + Triton with 10% FBS. Following washing, sections were incubated for 4 hrs. at room temperature with an Alexa Fluor 647 Donkey anti-Rabbit and Alexa Fluor 488 Donkey anti-Chicken (Invitrogen, A78948) secondary antibodies. Sections were mounted with SlowFade Glass Soft-set Antifade Mount. High Resolution imaging was completed using a Zeiss LSM900 Confocal Microscope.

All Antibodies and key reagents are referenced in **Supplemental Table 1.**

### Statistical Analysis.

All statistical analysis and graphing was completed using GraphPadPrism (10.4.1, 11.0.0). Data was analyzed using a one-way ANOVA followed by Tukey-Kramer post hoc analysis. Quantitative PCR was analyzed using nonparametric Wilcoxon/Kruskal-Wallis test and chi^2^ approximation.

## Supplementary Material

Supplementary Files

This is a list of supplementary files associated with this preprint. Click to download.

• 26513SupplementalInformation.pdf

• Cx43HumanWesternRAWGel.pdf

• 2654SUPPLEMENTALFIGURE2.tif

## Figures and Tables

**Figure 1:**
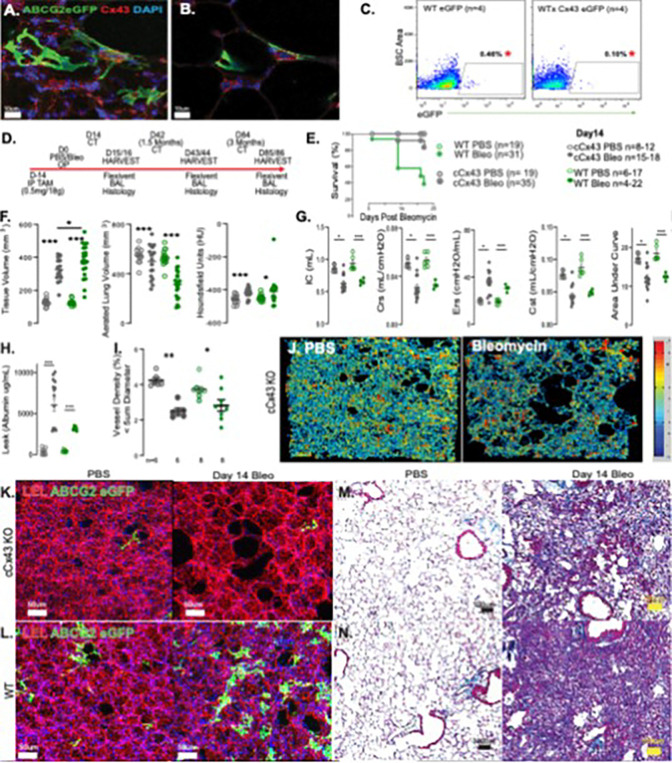
Heterozygous knockout of Cx43 in ABCG2 EPC drives an adaptive epithelial response to injury. Mice were induced with a single low dose of tamoxifen 2–3 day later lungs were harvested. **A.**Maximum intensity projection of immunostained vibratome sections from adult mouse lung to detect ABCG2-eGFP (green), Cx43 (red) and DAPI stained nuclei. **B.**Single slice through the z-stack. Scale=10μm. Immunostaining to detect Cx43 (red) in 3μm paraffin sectioned adult mouse lung tissue. Scale=20μm. **C.** Flow cytometric analysis of eGFP expression in pooled WT and cCx43KO mouse strains (n=4,4). **D.** Schematic of the experimental design. Mice were induced with a single low dose of tamoxifen, 2 weeks later vehicle or bleomycin was instilled o.p. and lung harvested on day-14 for endpoint analyses. **E.**Mean survival of mice at harvest, n per group and key. **F.** MicroCT endpoints. **G.** Airway physiological measurements taken using a Flexivent. **E.** Vascular leak quantitated by albumin present in the BAL. **H-J.**Quantitation of less than 5μm diameter vessel density from day-14 PBS vehicle or bleomycin injured vibratome sectioned mouse lung. n=8,8,6,6. Nonparametric one way Anova was used to define significance, *p<0.05, ** p<0.01, *** p<0.001. **J.** Representative density maps based on vessel diameter. **K,L.** Representative cCx43KO and WT maximum intensity projections of LEL (red) and ABCG2 eGFP labeled vibratome sections or **M,N.** trichrome stained mouse lung sections from day-14 PBS vehicle or bleomycin injured vibratome sectioned mouse lung.

**Figure 2:**
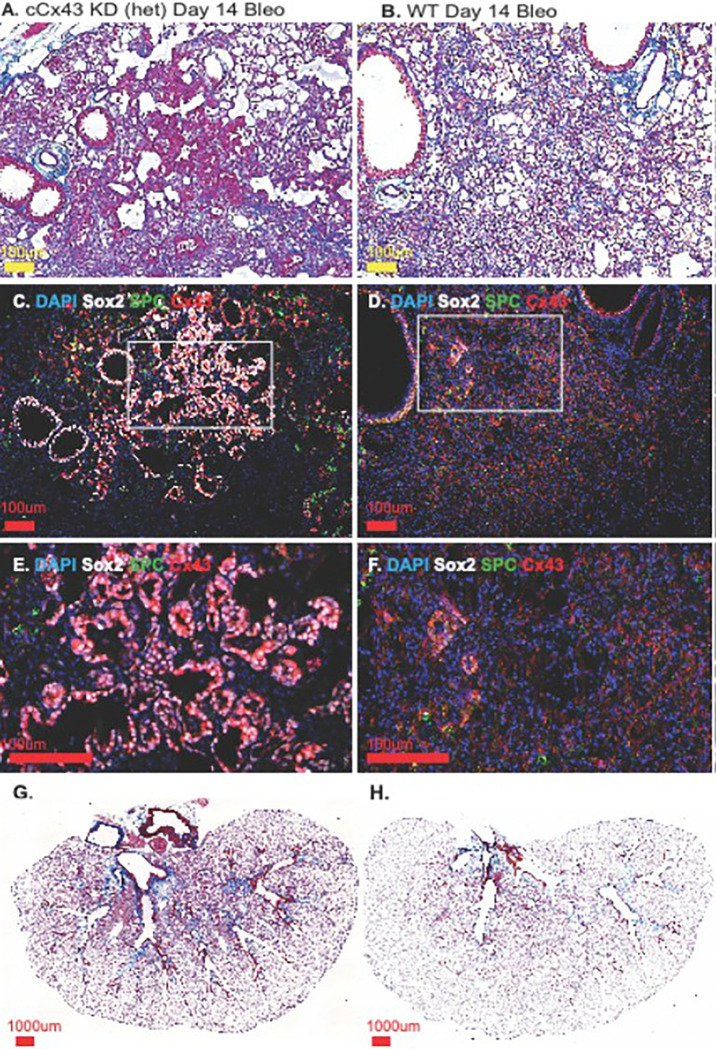
Ectopic basal epithelial cell appearance the alveolus confirmed by Sox2 and SPC localization. Mice were induced with a single low dose tamoxifen, 2 weeks later PBS vehicle or bleomycin was instilled o.p. and mice harvested on day-14 for histological analysis. Representative images of cCx43KO (**A,C,E**) and WT (**B,D,F**) mouse lung tissue sections. Scale =100μm. **A&B.**Trichrome staining. **C-F.** Immunofluorescent detection was performed to localize SOX2 (white), SFTPC (green), Cx43 (red) and nuclei with DAPI. **E&F.**Enlarged panels from C&D. **G&H.** Trichrome stained stitched images of cCx43KO or WT mouse lung sections from 3 month post bleomycin injury. Scale =1000μm.

**Figure 3:**
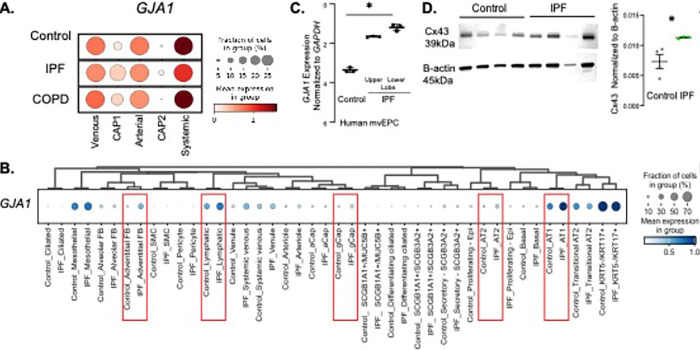
Increased expression of *GJA1* or Connexin 43 in human and mouse fibrosis. Dot plot of endothelial cells expressing *GJA1* in **A.** Control, IPF, and COPD (GSE136831) or **B.** Control and IPF non hematopoietic lung cells (GSE227136). Circle size represents the fraction of cells expressing *GJA1*, and color indicates mean expression level. Human control or IPF primary ABCG2^pos^ EPC **C.** expression level of *GJA1* relative to *GAPDH* (n=3) or **D.** Cx43 protein levels in control or IPF primary ABCG2^hi^ EPC relative to B-actin (n=4,4). Unpaired Welch’s t-test was used to define significance, *p<0.05, ** p<0.01, *** p<0.001.

## Data Availability

Data generated will be available upon request. Publicly available datasets: Gene Expression Omnibus (GEO)/NCBI: Human data: GSE227136 and GSE136831. This work was funded by grants to SMM: NHLBI R35HL161238, NIA RAG073317; PG and BWR: NIA RAG073317.
